# cIAP1 promotes proliferation and migration and prevents apoptosis in gallbladder cancer *in vitro*

**DOI:** 10.1042/BSR20182266

**Published:** 2019-04-12

**Authors:** Wei Su, Xiaojie Jiang, Mingyuan Chen, Maotuan Huang, Nanhong Tang, Xiaoqian Wang, Xiujin Li, Feifei She, Yanlin Chen

**Affiliations:** 1 Department of Hepatobiliary Surgery and Fujian Institute of Hepatobiliary Surgery, Fujian Medical University Union Hospital, Fujian Medical University, Fuzhou, China; 2 Key Laboratory of Ministry of Education for Gastrointestinal Cancer and Key Laboratory of Tumor Microbiology, The School of Basic Medical Sciences, Fujian Medical University, Fuzhou, China

**Keywords:** apoptosis, cellular inhibitor of apoptosis 1, gallbladder cancer, migration, nuclear factor-κB, proliferation

## Abstract

Gallbladder cancer (GBC) is a demanding fatal disease with no ideal treatment for inoperable patients. Recent reports have determined TNF-α associated lymphatic metastasis in GBC, while its resistance to TNF-α-killing remains largely unexplored. In this assay, we first found cellular inhibitor of apoptosis (cIAP1) overexpressed in GBC tissues and the roles in promoting the proliferation and migration of GBC *in vitro* as its homology cIAP2 does. Then how GBC cell survives TNF-α toxicity and TNF-α-induced apoptosis first prevail as follows. The reduction in cIAP1 does not give rise to apoptosis even with the stimulation of TNF-α. Importantly, the loss of cIAP1 enhanced TNF-α/cycloheximide-induced apoptosis in higher activation statuses of Caspase-8, Caspase-3 without the induction of Complex Ⅱ. In response to TNF-α, the reduction in cIAP1 caused the suppression in nuclear factor-κB (NF-κB) pathway and inhibition of transcription of cell death regulator cellular FLICE-like Inhibitory Protein (c-FLIP) instead. To conclude, cIAP1 is an oncological protein abundant in GBC tissues, which enhances proliferation and immigration and blocks TNF-α from apoptosis through NF-κB pathway *in vitro*.

## Introduction

Gallbladder cancer (GBC) ranks the sixth highest incidence in digestion system cancer worldwide, characterized as one of the most demanding cancers [1]. Radical surgery is highly recommended for localized GBC and accepted as the only cure for GBC patients. For the rest, who are often diagnosed at advanced stage, even extended resection becomes inappropriate and marries a morality of 10% less with 5-year survival rate [2]. In stark contrast, candidate treatments such as chemotherapy keep the mainstay for those sufferers but turn out to be too pale in medical effect [3]. However, insights into certain oncological molecules, which is heated in both basic research and translational medicine, may fuel targeted therapies that overcome GBC in the future.

It is well acknowledged that oncological behaviors of a tumor are attributed to the overexpressed molecules and its interaction with its embedded microenvironment. One of the most well-investigated cytokines flooded in the tumor microenvironment is TNF-α, which is capable of embarking on two distinct pathways in deciding life and death of cell [4,5]. In some case, TNF-α binds to TNFR in the membrane to form Complex by recruiting cellular inhibitor of apoptosis 1/2 (cIAP1/2) to enhance the translocation of NF-κB subunits into the nucleus and promote the transcription of various cytokines and proteins that mediate the progression of cancer or in another aspect, rescue cells from the fatal threats. In the other case, TNF-α sets off apoptosis via inducing the formation of cytoplasmic Complex Ⅱ where Caspase-8 is cleaved into biologically functional ones, followed by activation of the Caspase cascade and proteolysis of the whole cell in the end.

IAP family, which were originally named and indicated as inhibitor of apoptosis proteins, have been claiming increasing roles in various cell activities recently [6,7]. cIAP1, an important member of IAPs, functions as the ubiquitination ligase, with the abilities to regulate apoptosis, necroptosis, proliferation, migration, autophagy, and immunity [8]. Till now, piles of researches reported that cIAP1 were ectopically expressed or had gene amplification in cancers such as cervical cancer and bladder cancer, which conferred either resistance to therapies or implication for inferior prognoses [9–12]. Among those, some researchers owed the induction of apoptosis by some certain reagents to the elimination of cIAP1 without giving supporting mechanism, let alone the dispute over the controversial roles of cIAP1 in apoptosis [13–16]. Thus, our curiosity is whether its elimination of cIAP1 would be potent to limit the progression or overcome the resistance to apoptosis in GBC.

Here, we speculated that cIAP1 overexpressed in GBC tissues and identified both the malignant and anti-apoptotic roles of cIAP1 in GBC cells. In unstimulated condition, knockdown of cIAP1 limited the proliferation and migration of GBC but failed to make apoptosis happen. But elimination of cIAP1 sensitizes GBC cells to TNF-α-induced death when translations are generally inhibited in GBC cells, which may result from the impairment of activation of the NF-κB pathway and transcription of anti-apoptotic protein cellular FLICE-like Inhibitory Protein (c-FLIP). In brief, we urged that cIAP1 could enhance the proliferation and migration of GBC and boost apoptosis in particular circumstances.

## Methods

### Tissue samples and cell lines

A total of 64 tissues confirmed as GBC or not by Clinicopathology Department were included in our study. Resections were collected from patients who signed an informed consent in advance. Two GBC cell lines, including NOZ and SGC-996, were cultured in DMEM with FBS (Gibco, Grand Island, NY, U.S.A.) at 15 and 12% proportions separately. NOZ was purchased from the Health Science Research Resources Bank in Japan) and SGC-996 from the Tumor Cytology Research Unit, Medical College of Tongji University in China.

### Immunohistochemistry

Immunohistochemistry was performed as previously described [17]. Primary antibodies against cIAP1 (1:400, CST) were incubated with the sections overnight at 4°C. Five random fields per section were captured under a light microscope by identical Exposure Value (Olympus, Tokyo, Japan). Mean Optical Density was employed for the evaluation of each section. Each section was scored by two pathologists independently.

### Quantitative real-time polymerase chain reaction

Cells were extracted for total RNA with Tranzol UP reagent (Transgene), which were reversed transcribed with the Revert Aid First Strand cDNA Synthesis Kit (Transgene). The forward primer for *BIRC2* gene was 5′-AGCACGATCTTGTCAGATTGG-3′ and corresponding reverse one was 5′-GGCGGGGAAAGTTGAATATGTA-3′. The primers for GAPDH were 5′-AGGGCTGCTTTTAACTCTGGT-3′ (forward) and 5′-TCTCGCTCCTGGAAGATGGTG-3′ (reverse). The primers of CFLAR were 5′-TCAAGGAGCAGGGACAAGTTA-3′ (forward) and 5′-GACAATGGGCATAGGGTGTTATC-3′ (reverse). PCR was performed with Fast Start Universal SYBR Green Master Mix (Roche, Basel, Switzerland), and the intensity of fluorescence was measured with the ABI 7500 Real-Time System (Applied Biosystems, Life Technologies). Each reaction was performed in triplicate in the condition as described previously [18]. GAPDH served as an internal control and the data were analyzed by the 2^−ΔΔ*C*^_t_ method.

### Small interference RNA, short hairpin RNA, and transfection

Three siRNA strings targeting human cIAP1 sequence (GenBank accession number: NM-001165) were bought from GenePharma (Shanghai, China). Transfection of siRNA was performed in OptiMEM medium (Life Technologies) using Lipofectamine 2000 (Life Technologies). The pair: CCUGUGAACUUCUACAGAAUTT and the converse base pair AUUCUGUAGAGUUCACAGGTT were chosen for its knockdown efficiency. Then, synthesized short hairpin RNA oligonucleotides targeting cIAP1 (LV-shcIAP1) and negative control short hairpin RNA oligonucleotides (LV-NC) were annealed and ligated into the pGLVH1/RFP/Puro plasmid. The lentiviral vectors were used to infect GBC cells with treatment of puromycin for 2 weeks to established stably infected cells.

### Cell proliferation assay and cell death assay

Both the proliferation rate and viability of certain cells was reflected by the Cell Counting Kit-8 (CCK-8) assay with the manufacturer’s instructions (Dojindo Laboratories, Kumamoto, Japan). Briefly, a total of 5 × 10^3^/well were placed into 96-well plates and each well was added with 10 μl CCK-8 solution reagent for 1.5 h by the end. The results were determined by a plate reader at 450 and 600 nm for the interval of time of 24, 36, 48, 60, and 72 h (Bio-Rad, Hercules, CA, U.S.A.).

In cell death assay, approximately 2 × 10^4^/well were seeded into each well and treated with cycloheximide (5 mg/ml), TNF-α (40 ng/ml)/DMSO, TNF-α (40 ng/ml)/cycloheximide (5 mg/ml) and TNF-α/cycloheximide/z-VAD (20 nM) for 6 h. The process was performed as previously described. TNF-α was purchased from PeproTech, U.S.A., DMSO from Solarbio, China, cycloheximide from Shanghai Bio Industry, pan-Caspase inhibitor z-VAD from Beyotime Biotechnology. All conditions were performed in triplicate.

### Cell migration assay

The cell migration ability was tested with Transwell chamber (24-well format) with 8-μm polycarbonate membranes (Millipore, Washington, DC, U.S.A.). Cells were seeded at 5 × 10^5^/well in upper chambers with serum-free DMEM and the lower chamber was filled with 15% fetal bovine serum. Incubated for 72 h, cells that passed through the membranes were fixed with paraformaldehyde and stained with Crystal Violet. The number of cells on the lower side of the membrane was counted in five random fields (400×). Each condition was repeated in triplicate.

### Western blotting and immunoprecipitation

Western blotting was run as previously described with certain modifications adjusted to the molecular weight of certain protein. Anti-human cIAP1, anti-human Caspase-8, cleaved anti-human Caspase-8, cleaved anti-human Caspase-3, cleaved anti-human PARP, anti-human c-FLIP, and anti-human NF-κB (p65) were all purchased from Cell Signaling Technology (Cambridge, U.K.). β-actin was used as loading control in the early stage while GAPDH was a better candidate whenever apoptosis may happen. Histone-3 was used as loading control for nuclear fractions. Mild lysate NP-40 (Beyotime, China) other than RIPA was applied to avoid unexpected apoptosis [19].

The cells were pre-treated with z-VAD to prevent further reaction and incubated with stimulation of TNF-α (40 ng/ml)/cycloheximide (5 mg/ml) for 6 h [20]. An amount of 1:50 anti-human Caspase-8 or the same amount of IgG antibody together with Protein A/G Plus-Agarose (Santa Cruz Biotechnology) were added within the reaction system to incubate the complexes overnight. The mixtures were washed for three times with NP-40 by centrifuging, resuspended with 1× loading buffer, and subscribed to SDS/PAGE blotting for interested proteins. Each experiment was repeated in triplicate.

### Cell apoptosis assay

The percentage of apoptotic cells was determined by flow cytometry. The cells were placed in a six-well plate with good viability and treated with cycloheximide, TNF-α/DMSO, TNF-α/cycloheximide at concentrations mentioned above for 6 h. Then cells were dissociated, washed, and bound to Annexin-V and PI according to the manufacturer’s instructions. The intensity of fluorescence was collected by C6 machine (B&D system). Each condition was repeated in triplicate.

### Statistical analysis

The data were shown as means ± S.D. GraphPad Prism V7.0 was used to produce column graphs and FlowJo v10 to analyze the percentages of apoptotic cells and produce dot plots. Data process were processed by SPSS software (version 22.0) (applying Fisher’s exact test for quantitative data, a *t* test for comparing means between two groups, and one-way analysis of variance for comparing means among multiple groups). *P*<0.05 was considered statistically significant.

## Results

### The expression of cIAP1 is up-regulated in GBC tissues

**Figure 1:**
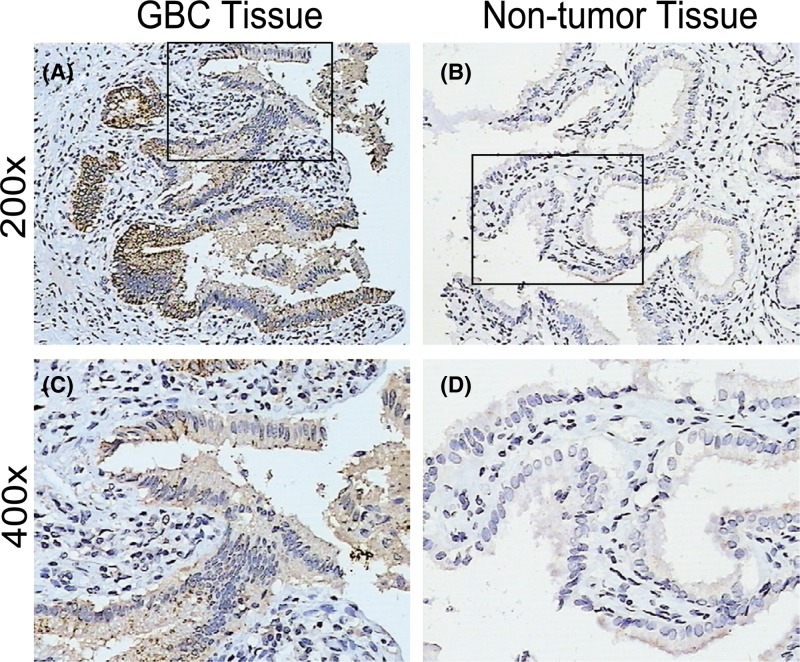
cIAP1 expression in GBC tissues is much stronger than non-tumor tissues (**A**) Representative glance of expression of cIAP1 in GBC tissues at 200×. (**B**) Representative glance of expression of cIAP1 in non-tumor tissues at 200×. (**C**) Magnified picture of hotspot from corresponding area in GBC tissue at 400×. (**D**) Magnified picture of hotspot from corresponding area in non-tumor tissues GBC tissue at 400×.

Without surprise, there were expressions ranging from moderate to strong in most GBC tissues (represented by Figure 1A,C) but non-tumor ones (represented by Figure 1B,D) presented weak or absent as compared. As shown by Table 1, GBC tissues were well stained with an averaged MOD of 0.2337, while the non-tumor ones were poorly stained with a mean MOD of 0.1205 as compared (*P*<0.001). Significantly, GBC tissues were characterized with elevated expression of cIAP1 in the cytoplasm and rarely located in the nucleus. As previously described, GBC is immersed in bile abundant with harmful TNF-α but simultaneously inherent with the abilities to migrate and invade. We wondered if the phenomenon had anything to do with overexpression of cIAP1.

**Table 1:** The expression of cIAP1 measured by MOD in GBC tissues and matched non-tumor tissues

	*n*	cIAP1 expression MOD (mean ± S.D.)	*t* value	*P*
GBC tissues	32	0.2337 ± 0.4785	9.214	<0.001
Non-tumor tissues	32	0.1205 ± 0.0504		

### Knockdown of cIAP1 limits the proliferation and immigration of GBC cells

**Figure 2:**
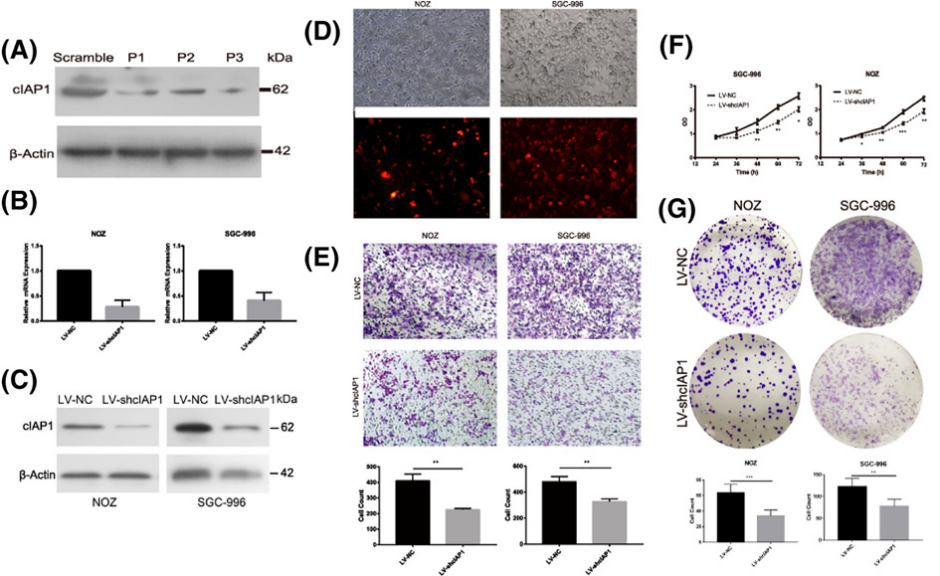
cIAP1 promotes the proliferation and migration of GBC *in vitro* (**A**) The knocking-down efficiency of three siRNA oligo strings were testified by Western blot. (**B**) The knockdown efficiencies were confirmed by quantitated PCR at mRNA level. (**C**) The knockdown efficiencies were confirmed by Western blotting at protein level. (**D**) Designate stably lentivirus-transfected cells including NOZ and SGC-996 were established as LV-shcIAP1 with controls of LV-NC. (**E**) Twice as much GBC migrated to the lower chamber in LV-NC than the LV-shcIAP1 (**P< 0.01 ). (**F**) A periodical test by CCK-8 reveal LV-shcIAP1 cell lines exhibited less viability and proliferation rate in 72 h. (**G**) Fewer clones were presented in LV-shcIAP1 group in clone formation assay (**P< 0.01,***P< 0. 001). All experiments were confirmed for at least three times.

We chose the most reliable siRNA strings out of three targeting cIAP1 by Western blotting (Figure 2A). Two stably transfected GBC cell lines, including NOZ and SGC-996, were established as LV-shcIAP1 carrying vectors expressing synthesized short hairpin RNA oligonucleotides or LV-NC with negative control ones. The transfected efficiencies were confirmed by comparing fluorescence scope with light one (Figure 2D) and knockdown efficiencies were assured both at mRNA and protein levels (Figure 2B,C). In Transwell migration assay, the number of GBC cells passing through the membrane in LV-shcIAP1 group counts twice as much than that of LV-NC at the end of 72 h (Figure 2E, *P*=0.003 in SGC-996 and *P*=0.0009 in NOZ), confirming cIAP1’s function in promoting migration of GBC *in vitro*. Compared with control group, LV-shcIAP1 became more lethargic and obtained a decreased growth rate in 3 days (Figure 2F) and in conformity, GBC cells in this group had inferior ability to form clones in 14 days (Figure 2G, *P*=0.004 in SGC-996 and *P*=0.002 in NOZ), indicating the loss of cIAP1 not only influenced short-term viability and proliferation but long-term proliferation in GBC.

### cIAP1 enhances TNF-α/cycloheximide-induced apoptosis in GBC cells

**Figure 3:**
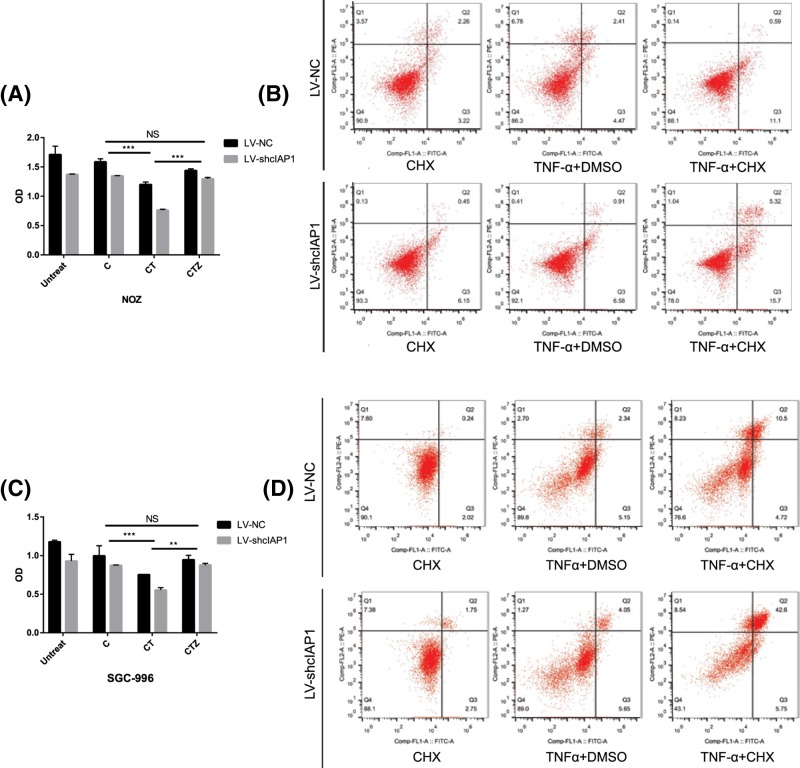
cIAP1 enhances TNF-α/cycloheximide-induced apoptosis in GBC (**A**,**C**) CCK-8 test at indicated time revealed co-administration of TNF-α (40 ng/ml) and cycloheximide (8 μg/ml) blunt cell viability instead of administration of TNF-α alone and the effect could be retrieved by 1 h pre-treatment by pan-Caspase inhibitor zVAD (20 mM). In each subgroup, the LV-shcIAP1 presented a lower viability and those with treatment of TNF-α and cycloheximide present the least (**P< 0.01, ***P< 0.001). (**B**,**D**) Similar groups were subscribed to flow cytometry for analysis of apoptosis. Apoptosis were observed obviously in TNF-α/cycloheximide group and even more ratio of apoptotic cells was observed in LV-shcIAP1 group. All the experiments were performed three times with similar results in both cell lines.

Although LV-shcIAP1 presented weakened viability as our previous studies showed, neither morphological change happened nor any differences on Caspase-3 blotting appeared, even with the presence of TNF-α in our preliminary experiment, indicating cIAP1 did not affect apoptosis directly. Then the GBCs, treated with the indicated doses of different combinations of stimulation, were observed for morphological change(s) with time using microscope (figures not shown) and the viabilities were determined in 6 h. The viability of GBC cells treated with cycloheximide, a pan-inhibitor of protein synthesis, showed no differences with that of the untreated group (Figure 3A,C). In comparison, the co-stimulation of TNF-α and cycloheximide reduced the viability of GBCs and the reduction was further set by knockdown of cIAP1. The effect could be retrieved by pan-Caspase inhibitor z-VAD compared with the group treated by cycloheximide, revealing it was a process that Caspases mediate. Additionally, knockdown of cIAP1 in each subgroups had decreased viability of GBC cells (*P*<0.001 in both cell lines). Then cells assigned with similar groups in six-well plates were stained with Annexin V/Propidium iodide and subscribed to flow cytometry to analyze apoptotic cells (Figure 3B,D). In accordance with previous results, no differences in apoptotic ratio were found between the LV-shcIAP1 and LV-NC in the presence/absence of TNF-α. The ratio of apoptotic cells was obviously increased in TNF-α and cycloheximide stimulated LV-NC cells, which doubled in the LV-shcIAP1 with identical stimulation. To summarize, knockdown of cIAP1 caused decreased viability and enhanced TNF-α/cycloheximide-induced apoptosis of GBC *in vitro*.

### cIAP1 sensitizes GBC cells to Caspase-8-associated apoptosis

**Figure 4:**
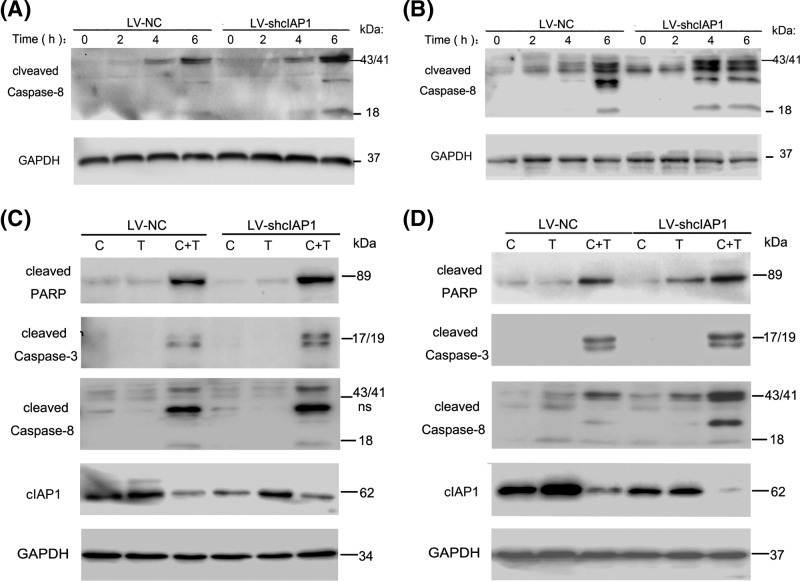
cIAP1 sensitizes GBC cells to Caspase-8-associated apoptosis (**A**,**B**) LV-shcIAP1 group have Caspase-8 cleaved and activated more readily and severely than LV-NC in periodical Western blot in 6 h. (**C**,**D**) Another arrangement of group of various combinations of stimulation were treated with two GBC cells with indicated dose and time. The group treating TNF-α/cycloheximide showed obvious decrease in cIAP1 and increase in cleaved PARP, cleaved Caspase-3, and cleaved Caspase-8. Proteins with proximate molecular weight were performed by separated gels in the same electrophoresis with loading control of GAPDH in triplicate.

It is reported that TNF-α could induce exogenous apoptosis by initially activating Caspase-8. Based on that, we figured out the effect of cIAP1 on cell death was associated with Caspase-8 in GBC cells stimulated with TNF-α/cycloheximide. It is suggested by Western blots that LV-shcIAP1 group presented a higher status of Caspase-8 activation than LV-NC did in a time-dependent way, which reached the peak at 4–6 h in SGC-996 and still increased after 6 h in NOZ (Figure 4A,B). Interested proteins including cleaved Caspase-8, cleaved Caspase-3, and cleaved PARP were analyzed for apoptosis in GBC cells. Despite the groups with cycloheximide or TNF-α/DMSO stimulation saw no distinct bands of those, concerning ones were well developed in the groups stimulated with TNF-α/cycloheximide. Consist with studies of CCK-8 and flow cytometry above, the quantitations of interested proteins in LV-shcIAP1 groups were generally twice as much than those of LV-NC groups, reassured our assumption that knockdown of cIAP1 made GBCs more sensitive to TNF-α/CHX-induced apoptosis via Caspase-8 activation. Interestingly, we found cIAP1 dramatically crushed down to a low level in response to TNF-α/cycloheximide while it was plain in SGC-996 and even elevated in NOZ in response to TNF-α (Figure 4C,D).

### cIAP1 inhibits apoptosis by NF-κB activation in GBC cells

**Figure 5:**
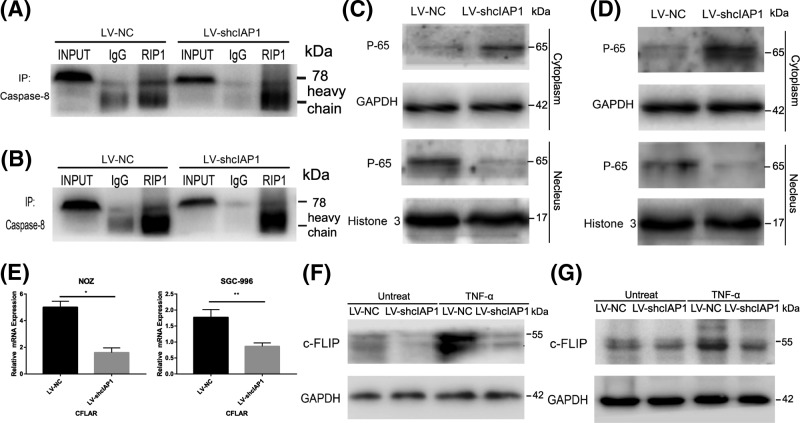
cIAP1 inhibits apoptosis by NF-κB activation in GBC (**A**,**B**) The immunoprecipitation analysis prior co-treatment of TNF-α and cycloheximide showed Caspase-8 with RIP1 but knockdown of cIAP1 failed to enhance such interaction with reference to Input ones. (**C**,**D**) There was less translocation of NF-κB subunit P65 from plasma to nuclear in LV-shcIAP1 group under the stimulation of TNF-α for 6 h in both cell lines. (**E**–**G**) The expressions of c-FLIP in both transcriptional and translational levels were reduced in LV-shcIAP1 group in the stimulation of TNF-α (*P< 0.05, **P< 0.01). Similar results were assured at least triplicated.

Apoptosis dominance by Caspase-8 was closely associated with the formation of Complex Ⅱ or degradation of Caspase-8-specific antagonist c-FLIP [21]. By immunoprecipitating with total Caspase-8, we found it bound to RIP1 but it is disappointing that this interaction was not altered by the loss of cIAP1 with reference to value of RIP1/Input (Figure 5A,B). However, with GBC cells exposed TNF-α for 6 h, a relatively less translocation of NF-κB subunit P65 from the cytoplasm to the nucleus, which is regarded as a golden standard of NF-κB activation was observed in LV-shcIAP1 cells (Figure 5C,D). Meanwhile, c-FLIP, of which the promoter located at NF-κB-binding sites, is reduced at the transcriptional level in LV-shcIAP1 cells (Figure 5E). As suggested by the bands, the protein increased when TNF-α is applied in LV-shcIAP1 cells but remained as much as both LV-NC and LV-shcIAP1 groups without stimulation (Figure 5F,G). In this part, we demonstrated that knockdown of cIAP1 influenced apoptosis by deactivating NF-κB pathway and down-regulated c-FLIP instead of enhancement of formation of Complex Ⅱ.

## Discussion

The relationship of GBC and its inflammatory microenvironment caused by gallstone has been long established and widely accepted [22]. TNF-α, which was flooded in the tumor environment, has been adapted into a pro-survival inflammatory factor promoting lymphangiogenesis regulated by RIP1 in GBC [23,24]. Of note, as a regulatory protein affiliated to RIP1, cIAP1 was thought to be the convertor to retrieve the trains of life from cliffs of death [25,26]. Here, we first observed the elevated cIAP1 expression in GBC tissues. Then, by stably infecting GBC cells with cIAP1 knocked down, we found cIAP1 was an important protein that claims the change in the proliferation and migration in GBC. Finally, we demonstrated cIAP1 protected GBC cells from TNF-α-induced killing by inducing the transcription of c-FLIP instead of an increasing assembly of RIP1-Caspase-8.

As previously demonstrated, cIAPs, including cIAP1 and cIAP2, are important components that claim pro-survival roles by ubiquitination on RIP1 in Complex I and anti-apoptotic one in preventing the formation of Complex Ⅱ [8]. As their functions seem quite overlapping, they each acquire different or unique biological roles. Consistent with our findings in cIAP1, cIAP2 promotes progression in GBC but their function in regulating apoptosis was hardly investigated [17]. There are unsettled controversies over the influence of cIAP1 alone or cIAPs upon apoptosis. Some researchers gave the assumption that cIAP1 was the essential protein affecting apoptosis independent of cIAP2, possibly for its outmatched proportion and function of degrading cIAP2. Physiologically, intestinal epithelium cells were sensitized to TNF-α-induced apoptosis by degradation of cIAP1 other than cIAP2 [27]. Oncologically, ovarian cancer cells were more readily responsive to the anticancer agent by knockdown of cIAP1 [28]. Pharmacologically, LCL16 knocked down both cIAP1 and cIAP2 to sensitize tumor to paclitaxel-induced cell death in colon cancer and specific antagonist inhibiting cIAP1 were potent to cause TNF-α-induced apoptosis in an autocrine way [29,30]. While the other scientists, which account for a majority, pointed out only by depletion of both cIAP1 and cIAP2 would apoptosis take place in the presence of TNF-α [21,31].

Our data were in conformity with the latter in which loss of cIAP1 did not directly induce apoptosis even with TNF-α stimulation in GBC cells, probably due to the protection of cIAP2 and constitutive activation of NF-κB in the cell itself [25,32]. Latter, we did unravel the loss of cIAP1 endowed GBC cells with a notably decreased viability and enhanced TNF-α/cycloheximide-induced apoptosis with a control administration of cycloheximide alone, which was supported by a more readily activated status of Caspase-8 and relevant proteins. Simultaneously, the changes in the level of cIAP1 and apoptosis coincided with the up-regulation of death regulatory proteins c-FLIP in both mRNA and protein levels, which were capable of suppressing the formation of RIP1/Caspase-8 mediated cell death [33]. Therefore, a step forward was taken to testify the indicated connection of cIAP1 and c-FLIP with the presence of TNF-α [34]. It turned out that knockdown of cIAP1 influences translocation of NF-κB p65 into the nucleus, followed by impairment on NF-κB activation, which was strongly supported by the conclusion that NF-κB inhibition induced apoptosis independent of cIAP2 [35]. Together, we hypothesized cIAP1, protects GBC cells from apoptosis by inducing transcription of c-FLIP mediated by NF-κB pathway. In addition, the impairment of NF-κB caused by the loss of cIAP1 may claim the decrease in proliferation and immigration in GBC cells due to the less transcription of some inflammatory cytokines as indicated [36]. Last, the level of cIAP1 was greatly descended within apoptotic GBC cells treated with TNF-α/cycloheximide compared with that of cycloheximide group but elevated to an extent in that of TNF-α group. It was probably the Caspases that were responsible for the sharp drop of the cIAP1 level and there was no literature proving the loss of cIAP1 could lead to apoptosis in positive feedback. While the rise of cIAP1 may answer why overexpression of cIAP1 co-exist with TNF-α and endure its toxicity *in vivo* and indirectly explain how this inflammatory cytokine promotes progression in GBC. In other words, there are plausible reasons supporting our assumption that aberrant cIAP1 expression and NF-κB hyperactivation make GBC cells free from the apoptosis induced by TNF-α by inducing the expression of c-FLIP.

Our study highlighted the oncological and protective roles of cIAP1 and the underlying mechanism inside in GBC cells *in vitro* for the first time. However, present work showed the loss of cIAP1 can fall short of TNF-α-killing in GBC cells due to the intervention of c-FLIP. Therefore, with respect to treatments by the cIAP1 elimination, the inhibitor of the NF-κB pathway or c-FLIP had better be utilized to realize higher effectiveness. As various IAP antagonists testing in different phases of clinical trials [37], our study may illuminate cIAP1 antagonists should be applied in combined with GBC injuring drugs such as cycloheximide to limit the progression and enhance apoptosis simultaneously.

## Compliance with ethical standards

All procedures performed in studies involving human participants were in accordance with the ethical standards of the institutional and/or national research committee and with the 1964 Helsinki Declaration and its later amendments or comparable ethical standards.

## Abbreviations

CCK-8: Cell Counting Kit-8
c-FLIP: cellular FLICE-like inhibitory protein
CHX: Cycloheximide
cIAP1: cellular inhibitor of apoptosis protein
GBC: gallbladder cancer
IAP: Inhibitor of Apoptosis Protein
MOD: Mean Optical Density
PARP: poly ADP-ribose polymerase
RIP1: Receptor Interaction Protein 1
TNFR: Tumor Necrosis Factor-Alpha Receptor
TNF-Alpha: Tumor Necrosis Factor-Alpha
