# Correction: cIAP1 promotes proliferation and migration and prevents apoptosis in gallbladder cancer in vitro

**DOI:** 10.1042/BSR-20182266_COR

**Published:** 2019-04-23

***Bioscience Reports* (2019) 39(4), BSR20182266; https://doi.org/10.1042/BSR20182266**

The authors of the published article have made an error in Figure 2. The correct Figure 2 and its legend is as follows:

**Figure 2:**
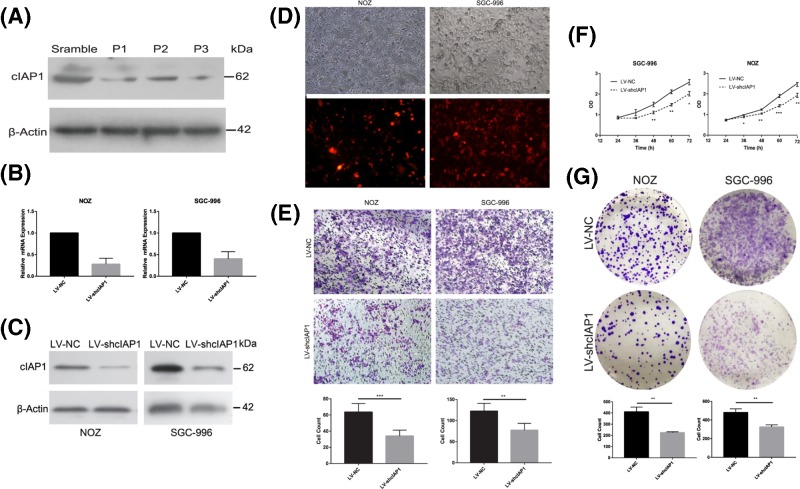
cIAP1 promotes the proliferation and migration of GBC in vitro (**A**) The knocking-down efficiency of three siRNA oligo strings were testified by Western blot. (**B**) The knockdown efficiencies were confirmed by quantitated PCR at mRNA level. (**C**) The knockdown efficiencies were confirmed by Western blotting at protein level. (**D**) Designate stably lentivirus-transfected cells including NOZ and SGC-996 were established as LV-shcIAP1 with controls of LV-NC. (**E**) Twice as much GBC migrated to the lower chamber in LV-NC than the LV-shcIAP1(**P< 0.01,***P< 0. 001). (**F**) A periodical test by CCK-8 reveal LV-shcIAP1 cell lines exhibited less viability and proliferation rate in 72 h. (**G**) Fewer clones were presented in LV-shcIAP1 group in clone formation assay (**P< 0.01).All experiments were confirmed for at least three times.

